# (1*S*,3*S*,4*S*)-*tert*-Butyl *N*-[1-benzyl-3-hydr­oxy-5-phenyl-4-(picolinamido)pent­yl]carbamate

**DOI:** 10.1107/S1600536808018965

**Published:** 2008-06-28

**Authors:** Jian-Feng Zheng, Su-Yu Huang, Jian-Nan Guo, Yu Zhang, Li-Ren Jin

**Affiliations:** a Department of Chemistry and Key Laboratory for Chemical Biology of Fujian Province, College of Chemistry and Chemical Engineering, Xiamen University, Xiamen, Fujian 361005, People’s Republic of China

## Abstract

The title compound, C_29_H_35_N_3_O_4_, was obtained by the reaction of (2*S*,4*S*,5*S*)-*tert*-butyl *N*-(4-amino-1-benzyl-3-hydr­oxy-5-phenyl­pent­yl)carbamate and picolinic acid using oxalyl chloride as a chlorinating reagent to activate the carboxyl group. In the crystal structure there are two mol­ecules in the asymmetric unit, which are aligned edge-to-face. In one mol­ecule, the pyridyl ring forms a dihedral angle of 22.0 (1)° with the phenyl ring of the terminal benzyl group and 14.3 (1)° with the other phenyl ring; in the other mol­ecule, the corresponding angles are 12.1 (1) and 10.6 (1)°, respectively. The packing is stabilized by inter­molecular hydrogen bonds and C—H⋯π inter­actions.

## Related literature

For related literature, see: Nishiyama *et al.* (1989 ▶); Allen *et al.* (1987 ▶); Pavel *et al.* (1993 ▶).
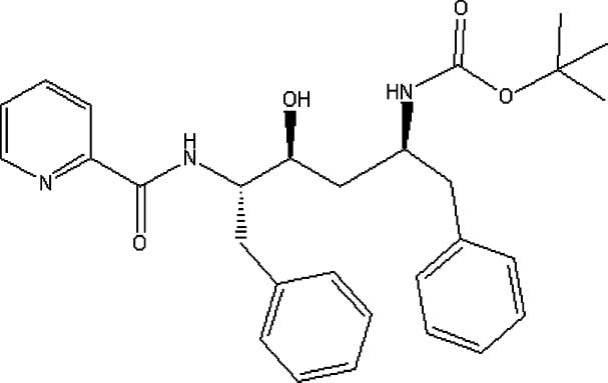


## Experimental

### 

#### Crystal data

C_29_H_35_N_3_O_4_
*M* *_r_* = 489.60
Monoclinic, 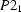

*a* = 11.7573 (12) Å
*b* = 15.9783 (18) Å
*c* = 15.0881 (15) Å
β = 103.787 (9)°
*V* = 2752.8 (5) Å^3^
*Z* = 4
Mo *K*α radiation
μ = 0.08 mm^−1^
*T* = 173 (2) K
0.70 × 0.32 × 0.12 mm

#### Data collection

Bruker APEX CCD diffractometer
Absorption correction: multi-scan (*SADABS*; Bruker, 2001 ▶) *T*_min_ = 0.947, *T*_max_ = 0.991
14395 measured reflections
4903 independent reflections
3396 reflections with *I* > 2σ(*I*)
*R*_int_ = 0.070

#### Refinement

*R*[*F* ^2^ > 2σ(*F* ^2^)] = 0.068
*wR*(*F* ^2^) = 0.180
*S* = 1.19
4903 reflections
649 parameters
1 restraint
H-atom parameters constrained
Δρ_max_ = 0.30 e Å^−3^
Δρ_min_ = −0.29 e Å^−3^

### 

Data collection: *SMART* (Bruker, 2001 ▶); cell refinement: *SAINT* (Bruker, 2001 ▶); data reduction: *SAINT*; program(s) used to solve structure: *SHELXS97* (Sheldrick, 2008 ▶); program(s) used to refine structure: *SHELXL97* (Sheldrick, 2008 ▶); molecular graphics: *ORTEP-3* (Farrugia, 1997 ▶); software used to prepare material for publication: *SHELXL97*.

## Supplementary Material

Crystal structure: contains datablocks I, global. DOI: 10.1107/S1600536808018965/cf2204sup1.cif

Structure factors: contains datablocks I. DOI: 10.1107/S1600536808018965/cf2204Isup2.hkl

Additional supplementary materials:  crystallographic information; 3D view; checkCIF report

## Figures and Tables

**Table 1:** Hydrogen-bond geometry (Å, °)

*D*—H⋯*A*	*D*—H	H⋯*A*	*D*⋯*A*	*D*—H⋯*A*
N2*A*—H2*AA*⋯O2*B*	0.88	2.04	2.888 (5)	162
O1*A*—H1*AB*⋯O3*B*	0.84	1.89	2.707 (5)	164
N2*B*—H2*BA*⋯O2*A*^i^	0.88	2.01	2.843 (5)	159
O1*B*—H1*BB*⋯O3*A*^i^	0.84	1.88	2.711 (5)	171
C23*B*—H23*B*⋯CgA^ii^	0.95	2.97	3.776 (4)	144

Symmetry codes: (i) 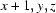; (ii) 
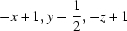
. *CgA* is the centroid of the C6–C11 phenyl ring.

**Fig. 1.**
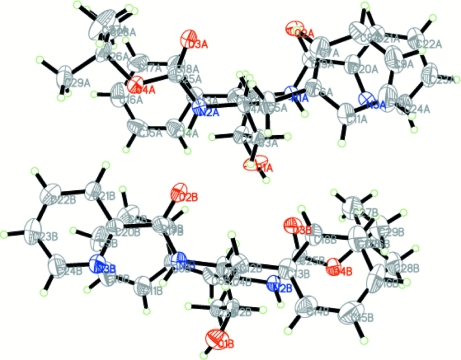
The asymmetric unit of (1) with the atom-labelling scheme, showing 50% probability displacement ellipsoids. H atoms are drawn as spheres of arbitrary radius.

**Fig. 2.**
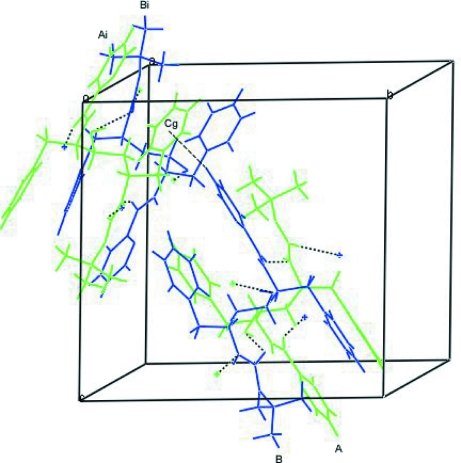
The packing of the molecules, viewed down the a axis. C—H···π interactions and hydrogen bonds are shown as dashed lines. Cg is the centroid of the C6/C11 phenyl ring.

## supplementary crystallographic information

### Comment

The title compound, (1), is a key intermediate for the preparation of recycling chiral ligands, such as oxazoline ligands for asymmetric catalysts. The majority of the syntheses of these ligands have followed a general synthetic route in which acids were first condensed with the corresponding optically active β-aminoalcohols to form the hydroxyamide derivatives. The hydoxy groups in the hydroxyamides were then activated and the resulting intermediates were cyclized to provide the oxazoline ligands (Nishiyama *et al.*, 1989).

The title compound was obtained by the reaction of (2*S*,4S,5*S*)-*tert*-butyl N-(4-amino-1-benzyl-3-hydroxy-5-phenylpentyl)carbamate and picolinic acid using oxalyl chloride as a chlorinating reagent to activate the carboxyl group. An X-ray crystal structure determination was carried out to determine its conformation. Bond lengths are in agreement with values reported in the literature (Allen *et al.*, 1987).

In the crystal structure of (1), there are two molecules in the asymmetric unit (Fig. 1). In molecule A, the dihedral angle of the pyridyl and the C6A/C11A phenyl rings is 22.0 (1)° and for the pyridyl and C13A/C18A rings it is 14.3 (1)°. In molecule B the angles are 12.1 (1)° and 10.6 (1)° respectively. The packing is shown in Fig. 2. Molecules are linked by O—H···O and N—H···O hydrogen bonds involving all the potential donors, generating linear chains parallel to the *a* axis. The packing is further stablized by C—H···π interactions, with typical geometry (Pavel *et al.*, 1993).

### Experimental

Picolinic acid (0.38 g, 3.12 mmol) was suspended in dichloromethane (40 ml) at 273 K. *N*,*N*-Dimethylformamide (0.1 ml) and oxalyl chloride (0.27 ml, 3.12 mmol) in dichloromethane (10 ml) were added and stirred for 3 h. The reaction mixture was then cooled to 263 K, (2*S*,4S,5*S*)-*tert*-butyl N-(4-amino-1-benzyl-3-hydroxy-5-phenylpentyl)carbamate (1.00 g, 2.60 mmol) and triethylamine (2.2 ml, 15.60 mmol) in dichloromethane (10 ml) were added dropwise and the mixture was stirred at 273 K. After 0.5 h, the reaction mixture was quenched with water (15 ml). The inorganic layer was separated followed by extraction with dichloromethane. The organic phases were combined and dried over anhydrous MgSO_4_ and concentrated *in vacuo*, and the residue was purified by silica gel column chromatography (ethyl acetate/petroleum ether 1:2), giving the product (1.89 g, 98.2%) as a white solid. Single crystals were obtained by slow evaporation of a solution in ethyl acetate/dichloromethane.

### Refinement

The hydrogen atoms were positioned geometrically (C—H = 0.93, 0.98, 0.97 or 0.96Å for phenyl, tertiary, methylene or methyl H atoms respectively) and were included in the refinement in the riding model approximation. The displacement parameters of methyl H atoms were set to 1.5*U*_eq_(C), while those of other H atoms were set to 1.2*U*_eq_(C). In the absence of significant anomalous scattering, Friedel pairs were merged; the absolute configuration was assumed from the synthesis.

### Crystal data

**Table:**

C_29_H_35_N_3_O_4_	*F*_000_ = 1048
*M**_r_* = 489.60	*D*_x_ = 1.181 Mg m^−^^3^
Monoclinic, *P*2_1_	Mo *K*α radiation λ = 0.71073 Å
Hall symbol: P 2yb	Cell parameters from 3695 reflections
*a* = 11.7573 (12) Å	θ = 4.1–32.7º
*b* = 15.9783 (18) Å	µ = 0.08 mm^−^^1^
*c* = 15.0881 (15) Å	*T* = 173 (2) K
β = 103.787 (9)º	Needle, colorless
*V* = 2752.8 (5) Å^3^	0.70 × 0.32 × 0.12 mm
*Z* = 4	

### Data collection

**Table:**

Bruker APEX CCD diffractometer	4903 independent reflections
Radiation source: fine-focus sealed tube	3396 reflections with *I* > 2σ(*I*)
Monochromator: graphite	*R*_int_ = 0.071
Detector resolution: 16.1903 pixels mm^-1^	θ_max_ = 26.0º
*T* = 173(2) K	θ_min_ = 4.1º
φ and ω scans	*h* = −13→14
Absorption correction: multi-scan(SADABS; Bruker, 2001)	*k* = −19→15
*T*_min_ = 0.947, *T*_max_ = 0.991	*l* = −18→18
14395 measured reflections	

### Refinement

**Table:**

Refinement on *F*^2^	Secondary atom site location: difference Fourier map
Least-squares matrix: full	Hydrogen site location: inferred from neighbouring sites
*R*[*F*^2^ > 2σ(*F*^2^)] = 0.068	H-atom parameters constrained
*wR*(*F*^2^) = 0.180	*w* = 1/[σ^2^(*F*_o_^2^) + (0.0854*P*)^2^] where *P* = (*F*_o_^2^ + 2*F*_c_^2^)/3
*S* = 1.19	(Δ/σ)_max_ = 0.175
4903 reflections	Δρ_max_ = 0.30 e Å^−^^3^
649 parameters	Δρ_min_ = −0.29 e Å^−^^3^
1 restraint	Extinction correction: none
Primary atom site location: structure-invariant direct methods	Absolute structure: indeterminate

### Special details

**Table:**

Geometry. All e.s.d.'s (except the e.s.d. in the dihedral angle between two l.s. planes) are estimated using the full covariance matrix. The cell e.s.d.'s are taken into account individually in the estimation of e.s.d.'s in distances, angles and torsion angles; correlations between e.s.d.'s in cell parameters are only used when they are defined by crystal symmetry. An approximate (isotropic) treatment of cell e.s.d.'s is used for estimating e.s.d.'s involving l.s. planes.
Refinement. Refinement of *F*^2^ against ALL reflections. The weighted *R*-factor *wR* and goodness of fit *S* are based on *F*^2^, conventional *R*-factors *R* are based on *F*, with *F* set to zero for negative *F*^2^. The threshold expression of *F*^2^ > σ(*F*^2^) is used only for calculating *R*-factors(gt) *etc*. and is not relevant to the choice of reflections for refinement. *R*-factors based on *F*^2^ are statistically about twice as large as those based on *F*, and *R*- factors based on ALL data will be even larger.

### Fractional atomic coordinates and isotropic or equivalent isotropic displacement parameters (Å^2^)

**Table:**

	*x*	*y*	*z*	*U*_iso_*/*U*_eq_	
N1A	0.2331 (3)	−0.0359 (3)	0.0924 (2)	0.0313 (10)	
H1AA	0.2848	−0.0193	0.0625	0.038*	
O1A	0.4696 (3)	−0.0739 (2)	0.1582 (3)	0.0478 (10)	
H1AB	0.5143	−0.0352	0.1500	0.072*	
C1A	0.2764 (4)	−0.0785 (3)	0.1767 (3)	0.0318 (12)	
H1AC	0.2258	−0.0636	0.2191	0.038*	
N1B	0.8118 (3)	0.0620 (3)	0.4204 (3)	0.0342 (11)	
H1BA	0.8836	0.0580	0.4545	0.041*	
O1B	0.9973 (3)	0.1330 (2)	0.3547 (2)	0.0479 (10)	
H1BB	1.0551	0.1006	0.3687	0.072*	
C1B	0.7958 (4)	0.1055 (3)	0.3347 (3)	0.0349 (13)	
H1BC	0.7262	0.0810	0.2911	0.042*	
N2A	0.3736 (3)	0.0516 (3)	0.3814 (2)	0.0348 (11)	
H2AA	0.4495	0.0452	0.4030	0.042*	
O2A	0.0392 (3)	−0.0395 (3)	0.0893 (2)	0.0478 (10)	
C2A	0.4011 (4)	−0.0460 (3)	0.2178 (3)	0.0333 (12)	
H2AB	0.4313	−0.0723	0.2790	0.040*	
N2B	0.8151 (3)	−0.0182 (3)	0.1264 (2)	0.0356 (11)	
H2BA	0.8743	−0.0222	0.1003	0.043*	
O2B	0.6237 (3)	0.0297 (3)	0.4112 (2)	0.0475 (10)	
C2B	0.9034 (4)	0.0912 (3)	0.2963 (3)	0.0316 (12)	
H2BB	0.8886	0.1187	0.2350	0.038*	
N3A	0.1977 (3)	0.0567 (3)	−0.0578 (3)	0.0422 (12)	
O3A	0.1966 (3)	0.0426 (3)	0.4110 (2)	0.0495 (10)	
C3A	0.4070 (4)	0.0499 (3)	0.2284 (3)	0.0316 (12)	
H3AB	0.4892	0.0665	0.2549	0.038*	
H3AC	0.3823	0.0759	0.1673	0.038*	
N3B	0.8810 (4)	−0.0150 (3)	0.5801 (3)	0.0378 (11)	
O3B	0.6284 (3)	0.0286 (3)	0.1105 (2)	0.0445 (10)	
C3B	0.9265 (4)	−0.0007 (3)	0.2838 (3)	0.0328 (12)	
H3BB	0.9978	−0.0057	0.2602	0.039*	
H3BC	0.9429	−0.0281	0.3444	0.039*	
O4A	0.3605 (3)	0.0011 (2)	0.5138 (2)	0.0419 (10)	
C4A	0.3306 (4)	0.0835 (3)	0.2884 (3)	0.0299 (12)	
H4AA	0.2496	0.0612	0.2644	0.036*	
O4B	0.7190 (3)	0.0316 (2)	−0.0065 (2)	0.0403 (10)	
C4B	0.8286 (4)	−0.0475 (3)	0.2206 (3)	0.0334 (12)	
H4BA	0.7543	−0.0348	0.2392	0.040*	
C5A	0.3234 (5)	0.1790 (3)	0.2860 (3)	0.0387 (14)	
H5AA	0.4037	0.2019	0.2959	0.046*	
H5AB	0.2905	0.1982	0.3371	0.046*	
C5B	0.8470 (5)	−0.1415 (3)	0.2263 (4)	0.0410 (14)	
H5BA	0.7871	−0.1687	0.1774	0.049*	
H5BB	0.9248	−0.1547	0.2154	0.049*	
C6A	0.2490 (4)	0.2143 (3)	0.1971 (3)	0.0332 (13)	
C6B	0.8397 (5)	−0.1770 (3)	0.3158 (4)	0.0420 (15)	
C7A	0.1329 (5)	0.1966 (4)	0.1733 (4)	0.0514 (17)	
H7AA	0.0993	0.1623	0.2117	0.062*	
C7B	0.7332 (5)	−0.1716 (4)	0.3433 (4)	0.0501 (16)	
H7BA	0.6661	−0.1476	0.3036	0.060*	
C8A	0.0632 (5)	0.2281 (4)	0.0934 (4)	0.0578 (19)	
H8AA	−0.0184	0.2166	0.0776	0.069*	
C8B	0.7267 (5)	−0.2011 (4)	0.4276 (4)	0.0528 (16)	
H8BA	0.6547	−0.1977	0.4456	0.063*	
C9A	0.1127 (6)	0.2765 (5)	0.0365 (4)	0.068 (2)	
H9AA	0.0665	0.2961	−0.0201	0.082*	
C9B	0.8228 (6)	−0.2352 (4)	0.4859 (4)	0.0584 (18)	
H9BA	0.8184	−0.2546	0.5445	0.070*	
C10A	0.2263 (6)	0.2954 (5)	0.0624 (5)	0.070 (2)	
H10A	0.2591	0.3319	0.0254	0.084*	
C10B	0.9243 (6)	−0.2409 (4)	0.4588 (4)	0.0608 (19)	
H10B	0.9900	−0.2675	0.4975	0.073*	
C11A	0.2988 (5)	0.2629 (4)	0.1423 (4)	0.0520 (17)	
H11A	0.3804	0.2744	0.1580	0.062*	
C11B	0.9343 (5)	−0.2095 (4)	0.3775 (4)	0.0481 (16)	
H11B	1.0086	−0.2101	0.3629	0.058*	
C12	0.2726 (5)	−0.1747 (3)	0.1633 (4)	0.0395 (14)	
H12A	0.1924	−0.1911	0.1304	0.047*	
H12B	0.3264	−0.1902	0.1246	0.047*	
C12B	0.7716 (5)	0.1984 (3)	0.3468 (3)	0.0363 (13)	
H12C	0.7017	0.2037	0.3721	0.044*	
H12D	0.8389	0.2228	0.3916	0.044*	
C13A	0.3058 (5)	−0.2229 (3)	0.2508 (4)	0.0370 (13)	
C13B	0.7517 (5)	0.2480 (3)	0.2595 (3)	0.0354 (13)	
C14A	0.4198 (5)	−0.2501 (4)	0.2874 (4)	0.0506 (17)	
H14A	0.4793	−0.2360	0.2571	0.061*	
C14B	0.8367 (5)	0.2968 (4)	0.2404 (4)	0.0475 (15)	
H14B	0.9105	0.2989	0.2831	0.057*	
C15A	0.4486 (5)	−0.2965 (4)	0.3657 (5)	0.0576 (19)	
H15A	0.5267	−0.3153	0.3886	0.069*	
C15B	0.8209 (6)	0.3437 (4)	0.1615 (5)	0.0633 (19)	
H15B	0.8827	0.3767	0.1495	0.076*	
C16A	0.3615 (6)	−0.3159 (4)	0.4118 (4)	0.0626 (19)	
H16A	0.3802	−0.3473	0.4669	0.075*	
C16B	0.7146 (6)	0.3412 (4)	0.1015 (4)	0.0562 (17)	
H16B	0.7023	0.3738	0.0474	0.067*	
C17A	0.2508 (6)	−0.2896 (5)	0.3770 (5)	0.072 (2)	
H17A	0.1912	−0.3029	0.4075	0.086*	
C17B	0.6229 (6)	0.2924 (4)	0.1168 (4)	0.0524 (17)	
H17B	0.5490	0.2915	0.0742	0.063*	
C18A	0.2234 (5)	−0.2434 (4)	0.2970 (4)	0.0528 (17)	
H18A	0.1449	−0.2255	0.2737	0.063*	
C18B	0.6428 (5)	0.2452 (3)	0.1963 (3)	0.0401 (14)	
H18B	0.5822	0.2106	0.2079	0.048*	
C19A	0.1186 (4)	−0.0190 (3)	0.0544 (3)	0.0348 (13)	
C19B	0.7258 (4)	0.0274 (3)	0.4519 (3)	0.0321 (12)	
C20A	0.1030 (5)	0.0318 (3)	−0.0288 (3)	0.0388 (13)	
C20B	0.7662 (4)	−0.0140 (3)	0.5421 (3)	0.0356 (13)	
C21A	−0.0087 (5)	0.0509 (4)	−0.0795 (4)	0.0460 (15)	
H21A	−0.0755	0.0336	−0.0592	0.055*	
C21B	0.6835 (5)	−0.0504 (4)	0.5833 (3)	0.0439 (15)	
H21B	0.6021	−0.0469	0.5558	0.053*	
C22A	−0.0218 (6)	0.0954 (5)	−0.1598 (4)	0.065 (2)	
H22A	−0.0976	0.1086	−0.1958	0.078*	
C22B	0.7245 (5)	−0.0917 (4)	0.6658 (4)	0.0492 (16)	
H22B	0.6712	−0.1186	0.6949	0.059*	
C23A	0.0758 (6)	0.1200 (4)	−0.1864 (4)	0.062 (2)	
H23A	0.0693	0.1503	−0.2416	0.075*	
C23B	0.8396 (6)	−0.0934 (4)	0.7039 (4)	0.0540 (17)	
H23B	0.8689	−0.1212	0.7604	0.065*	
C24A	0.1823 (6)	0.1005 (4)	−0.1331 (4)	0.0546 (17)	
H24A	0.2498	0.1198	−0.1513	0.066*	
C24B	0.9165 (5)	−0.0533 (4)	0.6590 (4)	0.0535 (17)	
H24B	0.9979	−0.0539	0.6870	0.064*	
C25A	0.3027 (4)	0.0322 (3)	0.4336 (3)	0.0289 (12)	
C25B	0.7128 (5)	0.0147 (3)	0.0796 (3)	0.0360 (13)	
C26A	0.2951 (5)	−0.0291 (4)	0.5801 (3)	0.0420 (14)	
C26B	0.6116 (4)	0.0549 (3)	−0.0752 (3)	0.0355 (13)	
C27A	0.2377 (7)	0.0439 (5)	0.6154 (5)	0.081 (2)	
H27A	0.1756	0.0666	0.5660	0.122*	
H27B	0.2963	0.0875	0.6376	0.122*	
H27C	0.2040	0.0252	0.6655	0.122*	
C27B	0.5242 (6)	−0.0143 (5)	−0.0854 (4)	0.066 (2)	
H27D	0.4934	−0.0173	−0.0305	0.099*	
H27E	0.5620	−0.0674	−0.0935	0.099*	
H27F	0.4597	−0.0035	−0.1387	0.099*	
C28A	0.2088 (6)	−0.0947 (5)	0.5389 (5)	0.064 (2)	
H28A	0.1457	−0.0694	0.4922	0.096*	
H28B	0.1759	−0.1200	0.5865	0.096*	
H28C	0.2479	−0.1379	0.5109	0.096*	
C28B	0.6612 (6)	0.0607 (4)	−0.1606 (4)	0.0573 (18)	
H28D	0.7183	0.1064	−0.1530	0.086*	
H28E	0.5972	0.0716	−0.2142	0.086*	
H28F	0.6995	0.0078	−0.1689	0.086*	
C29A	0.3931 (6)	−0.0683 (5)	0.6525 (4)	0.068 (2)	
H29A	0.4270	−0.1153	0.6258	0.102*	
H29B	0.3615	−0.0886	0.7031	0.102*	
H29C	0.4538	−0.0263	0.6752	0.102*	
C29B	0.5680 (6)	0.1377 (4)	−0.0535 (4)	0.0627 (19)	
H29D	0.5368	0.1329	0.0010	0.094*	
H29E	0.5058	0.1568	−0.1050	0.094*	
H29F	0.6324	0.1782	−0.0421	0.094*	

### Atomic displacement parameters (Å^2^)

**Table:**

	*U*^11^	*U*^22^	*U*^33^	*U*^12^	*U*^13^	*U*^23^
N1A	0.0250 (19)	0.039 (2)	0.030 (2)	0.0001 (19)	0.0074 (16)	0.0064 (19)
O1A	0.0418 (18)	0.041 (2)	0.072 (2)	−0.0081 (18)	0.0353 (17)	−0.0130 (18)
C1A	0.034 (2)	0.039 (3)	0.029 (2)	−0.007 (2)	0.0193 (19)	−0.006 (2)
N1B	0.033 (2)	0.038 (2)	0.031 (2)	0.003 (2)	0.0072 (17)	0.0077 (18)
O1B	0.046 (2)	0.037 (2)	0.056 (2)	−0.0089 (19)	0.0041 (17)	−0.0084 (19)
C1B	0.044 (3)	0.040 (3)	0.021 (2)	0.005 (2)	0.008 (2)	0.005 (2)
N2A	0.031 (2)	0.044 (3)	0.028 (2)	0.003 (2)	0.0037 (16)	0.0090 (19)
O2A	0.0287 (17)	0.072 (3)	0.0445 (19)	−0.0030 (19)	0.0115 (15)	0.012 (2)
C2A	0.046 (3)	0.028 (3)	0.030 (2)	0.003 (2)	0.017 (2)	0.002 (2)
N2B	0.033 (2)	0.049 (3)	0.024 (2)	−0.002 (2)	0.0053 (16)	0.0062 (19)
O2B	0.0439 (19)	0.067 (3)	0.0333 (18)	−0.007 (2)	0.0122 (15)	0.0144 (18)
C2B	0.035 (3)	0.033 (3)	0.025 (2)	−0.004 (2)	0.003 (2)	0.008 (2)
N3A	0.033 (2)	0.049 (3)	0.042 (2)	0.004 (2)	0.0058 (18)	0.010 (2)
O3A	0.041 (2)	0.069 (3)	0.0417 (19)	0.007 (2)	0.0155 (15)	0.0246 (19)
C3A	0.034 (2)	0.032 (3)	0.029 (2)	−0.005 (2)	0.0075 (19)	0.005 (2)
N3B	0.040 (2)	0.038 (3)	0.035 (2)	0.000 (2)	0.0076 (18)	0.0049 (19)
O3B	0.0330 (17)	0.063 (3)	0.0386 (18)	0.0011 (19)	0.0106 (15)	0.0039 (18)
C3B	0.038 (3)	0.036 (3)	0.027 (2)	0.001 (2)	0.012 (2)	0.000 (2)
O4A	0.0332 (17)	0.061 (3)	0.0329 (17)	−0.0042 (18)	0.0101 (14)	0.0114 (17)
C4A	0.033 (3)	0.030 (3)	0.024 (2)	0.004 (2)	0.002 (2)	0.005 (2)
O4B	0.0402 (18)	0.055 (2)	0.0234 (16)	−0.0034 (18)	0.0040 (14)	0.0030 (17)
C4B	0.041 (3)	0.028 (3)	0.032 (2)	0.000 (2)	0.009 (2)	0.003 (2)
C5A	0.058 (3)	0.032 (3)	0.025 (3)	0.003 (3)	0.008 (2)	−0.003 (2)
C5B	0.045 (3)	0.033 (3)	0.041 (3)	0.006 (3)	0.002 (2)	−0.003 (2)
C6A	0.039 (3)	0.031 (3)	0.033 (3)	0.009 (2)	0.015 (2)	0.003 (2)
C6B	0.056 (3)	0.031 (3)	0.036 (3)	−0.002 (3)	0.003 (2)	−0.001 (2)
C7A	0.055 (4)	0.052 (4)	0.049 (3)	0.003 (3)	0.016 (3)	0.007 (3)
C7B	0.047 (3)	0.043 (3)	0.059 (4)	−0.010 (3)	0.008 (3)	0.000 (3)
C8A	0.045 (3)	0.061 (4)	0.060 (4)	0.011 (3)	−0.004 (3)	0.005 (3)
C8B	0.060 (3)	0.043 (3)	0.064 (4)	−0.006 (3)	0.031 (3)	0.001 (3)
C9A	0.074 (4)	0.076 (5)	0.048 (4)	0.015 (4)	0.002 (3)	0.017 (4)
C9B	0.093 (5)	0.042 (4)	0.042 (3)	−0.012 (4)	0.019 (3)	0.008 (3)
C10A	0.070 (4)	0.070 (5)	0.062 (4)	−0.011 (4)	0.001 (3)	0.029 (4)
C10B	0.069 (4)	0.060 (4)	0.050 (4)	0.014 (4)	0.008 (3)	0.009 (3)
C11A	0.050 (3)	0.061 (4)	0.045 (3)	0.009 (3)	0.009 (3)	0.015 (3)
C11B	0.050 (3)	0.044 (3)	0.049 (3)	0.003 (3)	0.011 (3)	0.009 (3)
C12	0.044 (3)	0.035 (3)	0.044 (3)	−0.005 (3)	0.018 (2)	−0.004 (2)
C12B	0.040 (3)	0.032 (3)	0.036 (3)	0.008 (2)	0.005 (2)	−0.003 (2)
C13A	0.043 (3)	0.022 (3)	0.045 (3)	−0.003 (2)	0.009 (2)	0.000 (2)
C13B	0.047 (3)	0.025 (3)	0.037 (3)	0.007 (2)	0.014 (2)	−0.002 (2)
C14A	0.049 (3)	0.042 (3)	0.057 (4)	0.003 (3)	0.007 (3)	0.003 (3)
C14B	0.048 (3)	0.038 (3)	0.060 (3)	0.000 (3)	0.020 (3)	0.005 (3)
C15A	0.042 (3)	0.038 (3)	0.084 (5)	−0.004 (3)	−0.002 (3)	0.004 (3)
C15B	0.059 (4)	0.049 (4)	0.090 (5)	0.002 (3)	0.034 (3)	0.010 (4)
C16A	0.081 (5)	0.046 (4)	0.057 (4)	−0.016 (4)	0.009 (3)	0.010 (3)
C16B	0.095 (4)	0.036 (3)	0.048 (3)	0.011 (3)	0.039 (3)	0.012 (3)
C17A	0.058 (4)	0.071 (5)	0.094 (5)	−0.004 (4)	0.033 (4)	0.031 (4)
C17B	0.066 (4)	0.044 (3)	0.046 (3)	0.008 (3)	0.010 (3)	0.002 (3)
C18A	0.051 (3)	0.050 (4)	0.063 (4)	0.007 (3)	0.024 (3)	0.019 (3)
C18B	0.048 (3)	0.034 (3)	0.038 (3)	0.003 (3)	0.009 (2)	0.006 (2)
C19A	0.039 (3)	0.034 (3)	0.031 (3)	0.000 (3)	0.007 (2)	−0.008 (2)
C19B	0.029 (2)	0.036 (3)	0.032 (2)	0.004 (2)	0.010 (2)	−0.001 (2)
C20A	0.052 (3)	0.036 (3)	0.030 (2)	0.002 (3)	0.013 (2)	−0.001 (2)
C20B	0.033 (3)	0.037 (3)	0.037 (3)	0.002 (3)	0.009 (2)	−0.001 (2)
C21A	0.035 (3)	0.057 (4)	0.043 (3)	−0.001 (3)	0.004 (2)	0.012 (3)
C21B	0.047 (3)	0.051 (4)	0.037 (3)	0.006 (3)	0.017 (2)	0.012 (3)
C22A	0.058 (4)	0.079 (5)	0.052 (4)	0.005 (4)	0.000 (3)	0.022 (4)
C22B	0.056 (4)	0.046 (3)	0.049 (3)	−0.004 (3)	0.021 (3)	0.004 (3)
C23A	0.080 (4)	0.066 (5)	0.043 (3)	0.005 (4)	0.018 (3)	0.019 (3)
C23B	0.080 (4)	0.045 (3)	0.037 (3)	0.004 (3)	0.014 (3)	0.009 (3)
C24A	0.058 (3)	0.062 (4)	0.049 (3)	0.013 (3)	0.021 (3)	0.024 (3)
C24B	0.056 (4)	0.061 (4)	0.040 (3)	0.008 (3)	0.004 (3)	0.010 (3)
C25A	0.029 (2)	0.032 (3)	0.027 (2)	0.009 (2)	0.0085 (19)	0.001 (2)
C25B	0.041 (3)	0.034 (3)	0.033 (3)	−0.004 (3)	0.009 (2)	0.006 (2)
C26A	0.050 (3)	0.048 (3)	0.030 (3)	−0.018 (3)	0.014 (2)	0.006 (2)
C26B	0.035 (3)	0.034 (3)	0.030 (2)	−0.001 (3)	−0.008 (2)	0.008 (2)
C27A	0.122 (5)	0.081 (5)	0.062 (4)	−0.013 (5)	0.064 (4)	−0.007 (4)
C27B	0.069 (4)	0.062 (5)	0.061 (4)	−0.012 (4)	0.006 (3)	−0.002 (3)
C28A	0.072 (4)	0.058 (4)	0.057 (4)	−0.022 (4)	0.006 (3)	0.013 (3)
C28B	0.071 (4)	0.064 (4)	0.033 (3)	0.008 (4)	0.005 (3)	0.003 (3)
C29A	0.060 (4)	0.104 (6)	0.037 (3)	−0.018 (4)	0.003 (3)	0.025 (3)
C29B	0.075 (4)	0.056 (4)	0.056 (4)	0.010 (4)	0.013 (3)	0.007 (3)

### Geometric parameters (Å, °)

**Table:**

N1A—C19A	1.360 (6)	C10B—H10B	0.950
N1A—C1A	1.425 (6)	C11A—H11A	0.950
N1A—H1AA	0.880	C11B—H11B	0.950
O1A—C2A	1.414 (6)	C12—C13A	1.498 (7)
O1A—H1AB	0.840	C12—H12A	0.990
C1A—C2A	1.539 (7)	C12—H12B	0.990
C1A—C12	1.549 (7)	C12B—C13B	1.506 (7)
C1A—H1AC	1.000	C12B—H12C	0.990
N1B—C19B	1.336 (6)	C12B—H12D	0.990
N1B—C1B	1.440 (6)	C13A—C18A	1.361 (8)
N1B—H1BA	0.880	C13A—C14A	1.392 (8)
O1B—C2B	1.407 (6)	C13B—C14B	1.351 (8)
O1B—H1BB	0.840	C13B—C18B	1.404 (7)
C1B—C12B	1.531 (7)	C14A—C15A	1.367 (9)
C1B—C2B	1.529 (7)	C14A—H14A	0.950
C1B—H1BC	1.000	C14B—C15B	1.382 (9)
N2A—C25A	1.314 (6)	C14B—H14B	0.950
N2A—C4A	1.464 (6)	C15A—C16A	1.404 (10)
N2A—H2AA	0.880	C15A—H15A	0.950
O2A—C19A	1.220 (6)	C15B—C16B	1.358 (9)
C2A—C3A	1.542 (7)	C15B—H15B	0.950
C2A—H2AB	1.000	C16A—C17A	1.349 (9)
N2B—C25B	1.348 (6)	C16A—H16A	0.950
N2B—C4B	1.467 (6)	C16B—C17B	1.393 (9)
N2B—H2BA	0.880	C16B—H16B	0.950
O2B—C19B	1.212 (5)	C17A—C18A	1.387 (9)
C2B—C3B	1.513 (7)	C17A—H17A	0.950
C2B—H2BB	1.000	C17B—C18B	1.388 (8)
N3A—C24A	1.310 (7)	C17B—H17B	0.950
N3A—C20A	1.349 (7)	C18A—H18A	0.950
O3A—C25A	1.223 (6)	C18B—H18B	0.950
C3A—C4A	1.518 (7)	C19A—C20A	1.470 (7)
C3A—H3AB	0.990	C19B—C20B	1.486 (7)
C3A—H3AC	0.990	C20A—C21A	1.387 (7)
N3B—C24B	1.315 (7)	C20B—C21B	1.399 (8)
N3B—C20B	1.335 (6)	C21A—C22A	1.382 (8)
O3B—C25B	1.213 (6)	C21A—H21A	0.950
C3B—C4B	1.507 (7)	C21B—C22B	1.389 (8)
C3B—H3BB	0.990	C21B—H21B	0.950
C3B—H3BC	0.990	C22A—C23A	1.361 (9)
O4A—C25A	1.335 (5)	C22A—H22A	0.950
O4A—C26A	1.481 (6)	C22B—C23B	1.339 (9)
C4A—C5A	1.528 (7)	C22B—H22B	0.950
C4A—H4AA	1.000	C23A—C24A	1.354 (9)
O4B—C25B	1.346 (6)	C23A—H23A	0.950
O4B—C26B	1.478 (5)	C23B—C24B	1.406 (9)
C4B—C5B	1.518 (7)	C23B—H23B	0.950
C4B—H4BA	1.000	C24A—H24A	0.950
C5A—C6A	1.525 (7)	C24B—H24B	0.950
C5A—H5AA	0.990	C26A—C28A	1.488 (8)
C5A—H5AB	0.990	C26A—C27A	1.507 (10)
C5B—C6B	1.485 (8)	C26A—C29A	1.520 (8)
C5B—H5BA	0.990	C26B—C29B	1.484 (9)
C5B—H5BB	0.990	C26B—C27B	1.493 (9)
C6A—C7A	1.356 (8)	C26B—C28B	1.538 (8)
C6A—C11A	1.363 (8)	C27A—H27A	0.980
C6B—C11B	1.371 (8)	C27A—H27B	0.980
C6B—C7B	1.413 (8)	C27A—H27C	0.980
C7A—C8A	1.381 (8)	C27B—H27D	0.980
C7A—H7AA	0.950	C27B—H27E	0.980
C7B—C8B	1.376 (9)	C27B—H27F	0.980
C7B—H7BA	0.950	C28A—H28A	0.980
C8A—C9A	1.382 (10)	C28A—H28B	0.980
C8A—H8AA	0.950	C28A—H28C	0.980
C8B—C9B	1.369 (9)	C28B—H28D	0.980
C8B—H8BA	0.950	C28B—H28E	0.980
C9A—C10A	1.334 (10)	C28B—H28F	0.980
C9A—H9AA	0.950	C29A—H29A	0.980
C9B—C10B	1.354 (9)	C29A—H29B	0.980
C9B—H9BA	0.950	C29A—H29C	0.980
C10A—C11A	1.400 (8)	C29B—H29D	0.980
C10A—H10A	0.950	C29B—H29E	0.980
C10B—C11B	1.355 (9)	C29B—H29F	0.980
			
C19A—N1A—C1A	125.4 (4)	C18A—C13A—C14A	117.2 (5)
C19A—N1A—H1AA	117.3	C18A—C13A—C12	120.4 (5)
C1A—N1A—H1AA	117.3	C14A—C13A—C12	122.3 (5)
C2A—O1A—H1AB	109.5	C14B—C13B—C18B	118.3 (5)
N1A—C1A—C2A	107.5 (4)	C14B—C13B—C12B	121.4 (5)
N1A—C1A—C12	111.3 (4)	C18B—C13B—C12B	120.3 (5)
C2A—C1A—C12	112.5 (4)	C15A—C14A—C13A	121.9 (6)
N1A—C1A—H1AC	108.5	C15A—C14A—H14A	119.0
C2A—C1A—H1AC	108.5	C13A—C14A—H14A	119.0
C12—C1A—H1AC	108.5	C13B—C14B—C15B	122.9 (6)
C19B—N1B—C1B	124.9 (4)	C13B—C14B—H14B	118.6
C19B—N1B—H1BA	117.6	C15B—C14B—H14B	118.6
C1B—N1B—H1BA	117.5	C14A—C15A—C16A	119.2 (6)
C2B—O1B—H1BB	109.5	C14A—C15A—H15A	120.4
N1B—C1B—C12B	110.4 (4)	C16A—C15A—H15A	120.4
N1B—C1B—C2B	109.0 (4)	C16B—C15B—C14B	118.1 (6)
C12B—C1B—C2B	112.6 (4)	C16B—C15B—H15B	120.9
N1B—C1B—H1BC	108.2	C14B—C15B—H15B	120.9
C12B—C1B—H1BC	108.2	C17A—C16A—C15A	119.2 (6)
C2B—C1B—H1BC	108.2	C17A—C16A—H16A	120.4
C25A—N2A—C4A	122.2 (4)	C15A—C16A—H16A	120.4
C25A—N2A—H2AA	118.9	C15B—C16B—C17B	122.1 (6)
C4A—N2A—H2AA	118.9	C15B—C16B—H16B	118.9
O1A—C2A—C3A	111.1 (4)	C17B—C16B—H16B	119.0
O1A—C2A—C1A	105.7 (4)	C16A—C17A—C18A	120.5 (7)
C3A—C2A—C1A	113.1 (4)	C16A—C17A—H17A	119.8
O1A—C2A—H2AB	108.9	C18A—C17A—H17A	119.7
C3A—C2A—H2AB	108.9	C18B—C17B—C16B	118.0 (5)
C1A—C2A—H2AB	108.9	C18B—C17B—H17B	121.0
C25B—N2B—C4B	120.6 (4)	C16B—C17B—H17B	121.0
C25B—N2B—H2BA	119.7	C13A—C18A—C17A	121.9 (6)
C4B—N2B—H2BA	119.7	C13A—C18A—H18A	119.1
O1B—C2B—C3B	113.6 (4)	C17A—C18A—H18A	119.1
O1B—C2B—C1B	106.7 (4)	C17B—C18B—C13B	120.5 (5)
C3B—C2B—C1B	112.4 (4)	C17B—C18B—H18B	119.7
O1B—C2B—H2BB	107.9	C13B—C18B—H18B	119.7
C3B—C2B—H2BB	107.9	O2A—C19A—N1A	123.1 (5)
C1B—C2B—H2BB	107.9	O2A—C19A—C20A	124.4 (5)
C24A—N3A—C20A	119.0 (5)	N1A—C19A—C20A	112.3 (5)
C4A—C3A—C2A	113.3 (4)	O2B—C19B—N1B	123.3 (4)
C4A—C3A—H3AB	108.9	O2B—C19B—C20B	122.7 (5)
C2A—C3A—H3AB	109.0	N1B—C19B—C20B	114.0 (4)
C4A—C3A—H3AC	108.9	N3A—C20A—C21A	120.2 (5)
C2A—C3A—H3AC	108.9	N3A—C20A—C19A	119.7 (4)
H3AB—C3A—H3AC	107.7	C21A—C20A—C19A	120.1 (5)
C24B—N3B—C20B	117.7 (5)	N3B—C20B—C21B	122.9 (5)
C4B—C3B—C2B	115.3 (4)	N3B—C20B—C19B	117.9 (4)
C4B—C3B—H3BB	108.5	C21B—C20B—C19B	119.2 (4)
C2B—C3B—H3BB	108.4	C22A—C21A—C20A	119.3 (6)
C4B—C3B—H3BC	108.4	C22A—C21A—H21A	120.3
C2B—C3B—H3BC	108.4	C20A—C21A—H21A	120.4
H3BB—C3B—H3BC	107.5	C22B—C21B—C20B	117.8 (5)
C25A—O4A—C26A	120.0 (4)	C22B—C21B—H21B	121.1
N2A—C4A—C3A	109.3 (4)	C20B—C21B—H21B	121.1
N2A—C4A—C5A	112.0 (4)	C23A—C22A—C21A	118.8 (6)
C3A—C4A—C5A	112.1 (4)	C23A—C22A—H22A	120.6
N2A—C4A—H4AA	107.8	C21A—C22A—H22A	120.6
C3A—C4A—H4AA	107.8	C23B—C22B—C21B	119.6 (6)
C5A—C4A—H4AA	107.8	C23B—C22B—H22B	120.2
C25B—O4B—C26B	119.6 (4)	C21B—C22B—H22B	120.2
N2B—C4B—C3B	110.2 (4)	C22A—C23A—C24A	119.0 (6)
N2B—C4B—C5B	110.6 (4)	C22A—C23A—H23A	120.5
C3B—C4B—C5B	112.3 (4)	C24A—C23A—H23A	120.5
N2B—C4B—H4BA	107.9	C22B—C23B—C24B	118.9 (5)
C3B—C4B—H4BA	107.9	C22B—C23B—H23B	120.6
C5B—C4B—H4BA	107.9	C24B—C23B—H23B	120.5
C4A—C5A—C6A	114.0 (4)	N3A—C24A—C23A	123.7 (6)
C4A—C5A—H5AA	108.7	N3A—C24A—H24A	118.1
C6A—C5A—H5AA	108.7	C23A—C24A—H24A	118.1
C4A—C5A—H5AB	108.8	N3B—C24B—C23B	123.1 (5)
C6A—C5A—H5AB	108.7	N3B—C24B—H24B	118.4
H5AA—C5A—H5AB	107.6	C23B—C24B—H24B	118.5
C6B—C5B—C4B	113.1 (4)	O3A—C25A—N2A	123.4 (4)
C6B—C5B—H5BA	108.9	O3A—C25A—O4A	124.7 (4)
C4B—C5B—H5BA	109.0	N2A—C25A—O4A	111.9 (4)
C6B—C5B—H5BB	109.0	O3B—C25B—O4B	124.9 (4)
C4B—C5B—H5BB	109.0	O3B—C25B—N2B	125.4 (4)
H5BA—C5B—H5BB	107.8	O4B—C25B—N2B	109.7 (4)
C7A—C6A—C11A	120.6 (5)	C28A—C26A—O4A	111.1 (4)
C7A—C6A—C5A	119.1 (5)	C28A—C26A—C27A	112.0 (5)
C11A—C6A—C5A	120.3 (5)	O4A—C26A—C27A	109.5 (5)
C11B—C6B—C7B	117.1 (5)	C28A—C26A—C29A	109.5 (5)
C11B—C6B—C5B	123.3 (5)	O4A—C26A—C29A	101.1 (4)
C7B—C6B—C5B	119.4 (5)	C27A—C26A—C29A	113.1 (5)
C6A—C7A—C8A	120.4 (6)	O4B—C26B—C29B	110.6 (4)
C6A—C7A—H7AA	119.8	O4B—C26B—C27B	109.7 (4)
C8A—C7A—H7AA	119.8	C29B—C26B—C27B	114.7 (5)
C8B—C7B—C6B	120.0 (5)	O4B—C26B—C28B	99.9 (4)
C8B—C7B—H7BA	120.0	C29B—C26B—C28B	110.6 (5)
C6B—C7B—H7BA	120.0	C27B—C26B—C28B	110.3 (5)
C7A—C8A—C9A	119.7 (6)	C26A—C27A—H27A	109.5
C7A—C8A—H8AA	120.2	C26A—C27A—H27B	109.5
C9A—C8A—H8AA	120.1	H27A—C27A—H27B	109.5
C9B—C8B—C7B	120.7 (6)	C26A—C27A—H27C	109.5
C9B—C8B—H8BA	119.6	H27A—C27A—H27C	109.5
C7B—C8B—H8BA	119.7	H27B—C27A—H27C	109.5
C10A—C9A—C8A	119.0 (6)	C26B—C27B—H27D	109.5
C10A—C9A—H9AA	120.5	C26B—C27B—H27E	109.5
C8A—C9A—H9AA	120.5	H27D—C27B—H27E	109.5
C10B—C9B—C8B	119.0 (6)	C26B—C27B—H27F	109.5
C10B—C9B—H9BA	120.5	H27D—C27B—H27F	109.5
C8B—C9B—H9BA	120.5	H27E—C27B—H27F	109.5
C9A—C10A—C11A	122.0 (7)	C26A—C28A—H28A	109.5
C9A—C10A—H10A	119.0	C26A—C28A—H28B	109.5
C11A—C10A—H10A	119.0	H28A—C28A—H28B	109.5
C9B—C10B—C11B	121.4 (6)	C26A—C28A—H28C	109.4
C9B—C10B—H10B	119.3	H28A—C28A—H28C	109.5
C11B—C10B—H10B	119.3	H28B—C28A—H28C	109.5
C6A—C11A—C10A	118.2 (6)	C26B—C28B—H28D	109.5
C6A—C11A—H11A	120.9	C26B—C28B—H28E	109.5
C10A—C11A—H11A	120.9	H28D—C28B—H28E	109.5
C10B—C11B—C6B	121.6 (6)	C26B—C28B—H28F	109.5
C10B—C11B—H11B	119.2	H28D—C28B—H28F	109.5
C6B—C11B—H11B	119.2	H28E—C28B—H28F	109.5
C13A—C12—C1A	113.7 (4)	C26A—C29A—H29A	109.5
C13A—C12—H12A	108.8	C26A—C29A—H29B	109.5
C1A—C12—H12A	108.8	H29A—C29A—H29B	109.5
C13A—C12—H12B	108.8	C26A—C29A—H29C	109.4
C1A—C12—H12B	108.8	H29A—C29A—H29C	109.5
H12A—C12—H12B	107.7	H29B—C29A—H29C	109.5
C13B—C12B—C1B	113.6 (4)	C26B—C29B—H29D	109.5
C13B—C12B—H12C	108.8	C26B—C29B—H29E	109.5
C1B—C12B—H12C	108.8	H29D—C29B—H29E	109.5
C13B—C12B—H12D	108.9	C26B—C29B—H29F	109.5
C1B—C12B—H12D	108.8	H29D—C29B—H29F	109.5
H12C—C12B—H12D	107.7	H29E—C29B—H29F	109.5
			
C19A—N1A—C1A—C2A	−151.1 (4)	C12—C13A—C14A—C15A	−177.8 (5)
C19A—N1A—C1A—C12	85.3 (6)	C18B—C13B—C14B—C15B	0.0 (9)
C19B—N1B—C1B—C12B	88.6 (6)	C12B—C13B—C14B—C15B	−179.4 (6)
C19B—N1B—C1B—C2B	−147.2 (5)	C13A—C14A—C15A—C16A	−1.3 (9)
N1A—C1A—C2A—O1A	−66.7 (5)	C13B—C14B—C15B—C16B	1.0 (10)
C12—C1A—C2A—O1A	56.2 (5)	C14A—C15A—C16A—C17A	1.1 (10)
N1A—C1A—C2A—C3A	55.1 (5)	C14B—C15B—C16B—C17B	−0.9 (10)
C12—C1A—C2A—C3A	178.0 (4)	C15A—C16A—C17A—C18A	−0.5 (11)
N1B—C1B—C2B—O1B	−67.8 (5)	C15B—C16B—C17B—C18B	−0.1 (9)
C12B—C1B—C2B—O1B	55.2 (5)	C14A—C13A—C18A—C17A	−0.2 (9)
N1B—C1B—C2B—C3B	57.5 (5)	C12—C13A—C18A—C17A	178.5 (6)
C12B—C1B—C2B—C3B	−179.6 (4)	C16A—C17A—C18A—C13A	0.1 (11)
O1A—C2A—C3A—C4A	177.4 (4)	C16B—C17B—C18B—C13B	1.0 (8)
C1A—C2A—C3A—C4A	58.7 (5)	C14B—C13B—C18B—C17B	−1.0 (8)
O1B—C2B—C3B—C4B	−179.2 (4)	C12B—C13B—C18B—C17B	178.3 (5)
C1B—C2B—C3B—C4B	59.4 (5)	C1A—N1A—C19A—O2A	−0.5 (8)
C25A—N2A—C4A—C3A	−144.4 (5)	C1A—N1A—C19A—C20A	175.2 (4)
C25A—N2A—C4A—C5A	90.8 (6)	C1B—N1B—C19B—O2B	−0.6 (8)
C2A—C3A—C4A—N2A	64.0 (5)	C1B—N1B—C19B—C20B	179.8 (4)
C2A—C3A—C4A—C5A	−171.2 (4)	C24A—N3A—C20A—C21A	1.5 (8)
C25B—N2B—C4B—C3B	−120.8 (5)	C24A—N3A—C20A—C19A	178.7 (5)
C25B—N2B—C4B—C5B	114.5 (5)	O2A—C19A—C20A—N3A	174.7 (5)
C2B—C3B—C4B—N2B	68.0 (5)	N1A—C19A—C20A—N3A	−0.9 (7)
C2B—C3B—C4B—C5B	−168.3 (4)	O2A—C19A—C20A—C21A	−8.1 (8)
N2A—C4A—C5A—C6A	−164.0 (4)	N1A—C19A—C20A—C21A	176.3 (5)
C3A—C4A—C5A—C6A	72.8 (6)	C24B—N3B—C20B—C21B	1.2 (8)
N2B—C4B—C5B—C6B	−169.6 (4)	C24B—N3B—C20B—C19B	−178.6 (5)
C3B—C4B—C5B—C6B	66.9 (6)	O2B—C19B—C20B—N3B	178.2 (5)
C4A—C5A—C6A—C7A	63.7 (7)	N1B—C19B—C20B—N3B	−2.1 (7)
C4A—C5A—C6A—C11A	−116.2 (6)	O2B—C19B—C20B—C21B	−1.5 (8)
C4B—C5B—C6B—C11B	−111.4 (6)	N1B—C19B—C20B—C21B	178.1 (5)
C4B—C5B—C6B—C7B	63.3 (7)	N3A—C20A—C21A—C22A	0.2 (9)
C11A—C6A—C7A—C8A	−0.5 (9)	C19A—C20A—C21A—C22A	−177.0 (6)
C5A—C6A—C7A—C8A	179.6 (5)	N3B—C20B—C21B—C22B	−2.4 (9)
C11B—C6B—C7B—C8B	−2.3 (8)	C19B—C20B—C21B—C22B	177.3 (5)
C5B—C6B—C7B—C8B	−177.3 (5)	C20A—C21A—C22A—C23A	−0.7 (10)
C6A—C7A—C8A—C9A	1.4 (10)	C20B—C21B—C22B—C23B	1.9 (9)
C6B—C7B—C8B—C9B	0.5 (9)	C21A—C22A—C23A—C24A	−0.4 (11)
C7A—C8A—C9A—C10A	−3.4 (11)	C21B—C22B—C23B—C24B	−0.2 (9)
C7B—C8B—C9B—C10B	−1.1 (9)	C20A—N3A—C24A—C23A	−2.7 (9)
C8A—C9A—C10A—C11A	4.5 (12)	C22A—C23A—C24A—N3A	2.2 (11)
C8B—C9B—C10B—C11B	3.6 (10)	C20B—N3B—C24B—C23B	0.6 (9)
C7A—C6A—C11A—C10A	1.5 (9)	C22B—C23B—C24B—N3B	−1.1 (10)
C5A—C6A—C11A—C10A	−178.6 (6)	C4A—N2A—C25A—O3A	−3.5 (8)
C9A—C10A—C11A—C6A	−3.5 (11)	C4A—N2A—C25A—O4A	177.3 (4)
C9B—C10B—C11B—C6B	−5.6 (10)	C26A—O4A—C25A—O3A	4.3 (7)
C7B—C6B—C11B—C10B	4.8 (9)	C26A—O4A—C25A—N2A	−176.4 (4)
C5B—C6B—C11B—C10B	179.6 (6)	C26B—O4B—C25B—O3B	−11.5 (8)
N1A—C1A—C12—C13A	−174.2 (4)	C26B—O4B—C25B—N2B	169.9 (4)
C2A—C1A—C12—C13A	65.0 (6)	C4B—N2B—C25B—O3B	5.5 (8)
N1B—C1B—C12B—C13B	−178.9 (4)	C4B—N2B—C25B—O4B	−175.9 (4)
C2B—C1B—C12B—C13B	59.0 (6)	C25A—O4A—C26A—C28A	56.5 (7)
C1A—C12—C13A—C18A	87.8 (6)	C25A—O4A—C26A—C27A	−67.7 (6)
C1A—C12—C13A—C14A	−93.6 (6)	C25A—O4A—C26A—C29A	172.7 (5)
C1B—C12B—C13B—C14B	−101.6 (6)	C25B—O4B—C26B—C29B	67.7 (6)
C1B—C12B—C13B—C18B	79.1 (6)	C25B—O4B—C26B—C27B	−59.8 (6)
C18A—C13A—C14A—C15A	0.9 (9)	C25B—O4B—C26B—C28B	−175.7 (5)

### Hydrogen-bond geometry (Å, °)

**Table:**

*D*—H···*A*	*D*—H	H···*A*	*D*···*A*	*D*—H···*A*
N2A—H2AA···O2B	0.88	2.04	2.888 (5)	162
O1A—H1AB···O3B	0.84	1.89	2.707 (5)	164
N2B—H2BA···O2A^i^	0.88	2.01	2.843 (5)	159
O1B—H1BB···O3A^i^	0.84	1.88	2.711 (5)	171
C23B—H23B···CgA^ii^	0.95	2.97	3.776 (4)	144

Symmetry codes: (i) *x*+1, *y*, *z*; (ii) −*x*+1, *y*−1/2, −*z*+1.
